# Bee-Associated Beneficial Microbes—Importance for Bees and for Humans

**DOI:** 10.3390/insects15060430

**Published:** 2024-06-06

**Authors:** Svetoslav Dimitrov Todorov, Marcos Vinício Alves, Gisana Cristina Alves Bueno, Virgínia Farias Alves, Iskra Vitanova Ivanova

**Affiliations:** 1 ProBacLab, Laboratório de Microbiologia de Alimentos, Departamento de Alimentos e Nutrição Experimental, Faculdade de Ciências Farmacêuticas, Universidade de São Paulo, São Paulo 05508-000, SP, Brazil; 2 CISAS-Center for Research and Development in Agrifood Systems and Sustainability, Instituto Politécnico de Viana do Castelo, 4900-347 Viana do Castelo, Portugal; 3 Faculdade de Farmácia, Universidade Federal de Goiás (UFG), Goiânia 74605-170, GO, Brazilvirginia_alves@ufg.br (V.F.A.); 4 Department of General and Industrial Microbiology, Faculty of Biology, Sofia University St. Kliment Ohridski, 8, Bul. Dragan Tzankov, 1164 Sofia, Bulgaria; iskrai3@yahoo.com

**Keywords:** bees, microbiota, fructophilic bacteria, probiotics, bacteriocins

## Abstract

**Simple Summary:**

Bees play an essential role in maintaining biodiversity, as they are crucial for the pollination provisions of agricultural crops and for plants in general. In recent years, a decline in bee populations has been noted, especially due to climatic and anthropometric factors and challenges. It is important to develop (or redevelop) approaches that can help preserve these important insects. Improving our knowledge on bees’ microbiology can be one of the ways to better understand ecological relations and the life cycle of these beneficial tiny workers. One of the possible alternatives to overcome the decline in bee populations, while improving their health and productivity, is to study their microbiota, and that of their related products, and identify beneficial microorganisms that may be useful for use as probiotics. The microorganisms present in bees’ microbiota make an essential contribution to their health by being involved in metabolism and providing food supplies, helping to digest and preserve food, and protecting them from the diseases. In this review, we highlight some of the main bacterial representatives of the microbiota of different species of bees and their by-products, with the focus on microorganisms with characteristics improving health, and other possible applications in the food industry. At the end, “One Health” is not just a book concept, but a real scientific strategy.

**Abstract:**

Bees are one of the best-known and, at the same time, perhaps the most enigmatic insects on our planet, known for their organization and social structure, being essential for the pollination of agricultural crops and several other plants, playing an essential role in food production and the balance of ecosystems, being associated with the production of high-value-added inputs, and a unique universe in relation to bees’ microbiota. In this review, we summarize information regarding on different varieties of bees, with emphasis on their specificity related to microbial variations. Noteworthy are fructophilic bacteria, a lesser-known bacterial group, which use fructose fermentation as their main source of energy, with some strains being closely related to bees’ health status. The beneficial properties of fructophilic bacteria may be extendable to humans and other animals as probiotics. In addition, their biotechnological potential may ease the development of new-generation antimicrobials with applications in biopreservation. The concept of “One Health” brings together fundamental and applied research with the aim of clarifying that the connections between the different components of ecosystems must be considered part of a mega-structure, with bees being an iconic example in that the healthy functionality of their microbiota is directly and indirectly related to agricultural production, bee health, quality of bee products, and the functional prosperity for humans and other animals. In fact, good health of bees is clearly related to the stable functionality of ecosystems and indirectly relates to humans’ wellbeing, a concept of the “One Health”.

## 1. Introduction

Since Metchnikoff and his Bulgarian collaborator Stamen Grigorov [1] postulated health benefits from consumption of yoghurt and suggested that these advantages may be related to the lactic acid bacteria (LAB) responsible for the fermentation process, research focused on different LAB and the exploration of their favorable properties has grown exponentially. Several research projects have highlighted the potential and actual probiotic properties of LAB [2], and detailed mechanisms involved in immunostimulatory activity, competitive exclusion, production of beneficial metabolites, etc. [3]. The beneficial role of bifidobacteria has been suggested as well [4], and they, together with LAB, have become the main studied probiotic microorganisms with application in humans, pets, and farming animals. Moreover, in the last decade, there has been special focus on so-called NGPs (New-Generation Probiotics), where beneficial properties of microbial cultures other than LAB and bifidobacteria have been suggested and explored [5]. The concept, based on built knowledge regarding the role of different microorganisms in gastrointestinal tract (GIT) balance, has served as scientific inducement for the evaluation of the beneficial properties of different microorganisms in the context of the complexity of humans and other animals. All this has stimulated the search for new sources of beneficial microorganisms that can be applied in different areas, such as food/feed and health for humans and other living creatures.

Bees of different species contribute directly to plant pollination, being crucial for maintaining healthy ecosystems and global agriculture. The GIT of bees is colonized by symbiont microorganisms, which are associated mainly with flowers (pollinated by bees) and contact between bees, but also from the surrounding ecosystem, and these symbionts actively contribute to the health and productivity of bees [6]. These microorganisms (generally bacterial species) are also present in beehive inputs, such as honey, bee-collected pollen, and bee bread, contributing to these foods being an invaluable source of bioactive compounds which not only support the health of the hives, but also promote nutrition, health, and consumers’ well-being, being considered functional foods [6,7]. In addition to beneficial microorganisms, several bacterial species have also been identified in bees and hives (as part of the complex microbiota) that have an adverse effect on the health of bees—*Paenibacillus larvae*, *Melissococcus plutonius*, *Spiroplasma apis*, and *Spiroplasma melliferum*, as well as fungi, such as *Aspergillus*, *Nosema*, and *Varroa* species, besides many viruses and protozoa [8,9,10]. Studies on the microbiome of bees are of great importance to improve our understanding of their biology, as, in addition to reducing the negative impacts of undesired microbes on bee health and productivity, they can also help us mitigate environmental risks that these important pollinated animals have suffered.

**Figure 1:**
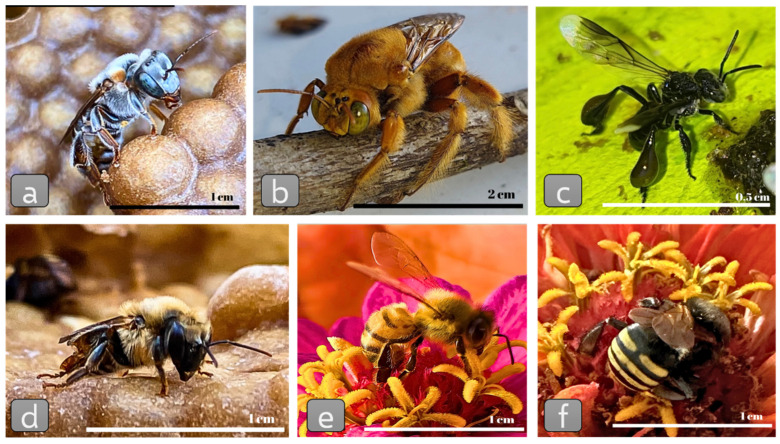
Different species of bees: (**a**) stingless bee *Melipona fasciculata*; (**b**) male solitary bee *Xylocopa* sp.; (**c**) stingless bee *Friseomielita languida*; (**d**) stingless bee *Melipona quadrifasciata anthidioides*; (**e**) *Apis mellifera* pollinating a flower; (**f**) stingless bee *Melipona quadrifasciata anthidioides* pollinating a flower.

Currently, around 20,000 bee species have been described worldwide (some examples are presented on Figure 1), with remarkable morphological, ecological, and behavioral diversity, but only a small percentage of these species have been studied at the microbiome/microbiota level [11,12]. However, this scenario is likely to change soon, as the tools for studying microorganisms have become more powerful, allowing us to better understand microbial ecosystems. Approaches based on high-throughput DNA sequencing have allowed a more reliable image for the microbial ecology of bees. However, a combination of different experimental approaches is still an optimal realistic experimental research scenario.

In this manuscript, we will provide an update on the knowledge regarding the microbiota of the honeybee and some other less-known bee species, highlighting the functionality of bacterial groups of biotechnological interest, focusing on microorganisms with beneficial/probiotic potential.

## 2. The Microbiota of Western Honeybees (*Apis mellifera*)

Bees are insects with economical and agricultural importance, not only due to production of honey, but much more associated with the pollination of crops, being thus related to food supply security. Despite the importance of these insects in nature and maintenance of ecological balance, their populations have declined in the last decade and are still declining worldwide mainly due to Colony Collapse Disorder (CCD) [13]. To understand and prevent this disorder, microbial interactions—both symbiotic and pathogenic—have been investigated [14,15].

The Western honeybee, also referred to as the European honeybee, *Apis mellifera*, is the most investigated bee species due to its importance as a pollinator of agricultural crops around the world. Moreover, the species has undergone substantial domestication, being currently responsible for producing several inputs (e.g., honey, wax, fermented pollen) that are traded as international commodities with high added value. Throughout its native distribution, *A. mellifera* exhibits substantial genetic and phenotypic variation in behavioral and morphological characteristics, with distinct lineages and subspecies described based on geometric, morphometric, and genetic studies [16], which is beyond the scope of this study.

*A. mellifera* is considered a good model for the study of microbiota, as its microbiome consists of a small subset of phylotypes and associated species of fungi, viruses, and bacteria [8]. However, the knowledge of the microbiota associated with honeybees is still limited, as are varieties between different life stages (egg, larva, pupa, adult) and castes (drones, queens, workers), among the ecological niches present in hives (e.g., honeycombs, brood combs, pathosphere) and the availability and nutrient content of flowering plants [8,17].

Studies based on 16S rRNA sequences and metagenomic research on adult *A. mellifera*, regardless of their geographic origin, life stage, or season turnover, indicate that the main host-adapted bacterial phyla present in their gut microbiota comprise Proteobacteria, Firmicutes, Actinobacteria, and Bacteroidetes, with lower frequencies of phyla such as Cyanobacteria, Verrucomicrobia, Acidobacteria, Chloroflexi, Spirochaetes, and Planctomycetes [18,19]. Although there are short-term shifts in the microbial taxa present in *A. mellifera*, it has been described that the adult honeybee’s microbiota is dominated (up to 99.9%) by 9–10 bee-associated bacterial clusters, each representing a complex of related strains that are transmitted through social interactions between individuals, with five species’ clusters forming the main core of the bee gut community, albeit with different relative abundances differing among studies [19,20,21,22]. Principle reported ubiquitous Gram-negative species were *Snodgrassella alvi* (phylum Pseudomonadota) and *Gilliamella apicola* (phylum Proteobacteria), while ubiquitous and abundant Gram-positive species were represented by two species clusters, namely, *Lactobacillus mellifer*/*mellis* clade (formerly Firm-4 clade) and *Lactobacillus melliventris* clade (formerly Firm-5 clade), both from Firmicutes phylum, with *Bifidobacterium asteroides*-related species cluster (phylum Actinobacteria) also being present in most adult bees [20,22,23,24,25]. Additional phylotypes that are less consistent across colonies (non-core) and are not necessarily present in every individual include the *Frischella*, *Bartonella*, *Commensalibacter*, *Bombella*, *Fructobacillus* and *Apibacter* species [6,22,23,26,27]. It is also worth mentioning *Apilactobacillus kunkeei* (previously referred to as *Lactobacillus kunkeei*), a non-core colonizer species that accounts for a relatively small proportion of the honeybee gut microbiota, although it is quite common in the floriferous environment and in bees’ inputs [6,21,23,25]. All further abbreviations for the former *Lactobacillus* genera are stated according to Todorov et al. [28].

Microbial communities have been shown to change throughout the life cycle of bees, where gut colonization is changed over bee age [29]. In larvae and newborns, bees are almost microbe-free in the gut, presenting a relatively stable simple microbial community at around nine days of age [30]. Worker bees and queens share many essential gut bacterial species, and although not every species present in the forager is necessarily present in the queen, species such as *Apb. kunkeei* and *Bombella apis* (formerly *Parasaccharibacter apium*) are common species in the queen’s gut [31,32]. The GIT in adult honeybees is divided into four parts: stomach (crop), middle throat, ileum, and rectum [22]. Each compartment has a distinct environment favoring specific microorganisms, and thus there are variations in the microbial communities in different parts of a bee’s GIT, with the stomachs and mid-intestines of foragers being almost devoid of bacteria, while their ileum and rectum contain most of the total bacterial biomass [8,22].

Over the course of their lives, worker bees perform many different tasks that can contribute to these variations. Newly emerged worker bees build and maintain the wax combs, protect the colony, and receive and process food from the pollinating bees [33]. They are specialized workers, and their job is to collect nectar and pollen from different flowers during the season. Honeybee foragers collect and bring the pollen to the hive (bee-pollen), where it is mixed with glandular secretions, nectar, or honey, processed by symbiont microbes, and becomes bee bread, the naturally fermented pollen that is colonies’ staple food [34]. Bee bread is a highly nutritious food that contains large bacterial diversity, some of which is crucial for the performance of the fermentation process conducting transformation of pollen (plant material) into a more digestible form for bees, and further contributing not only to their nutritional needs, but providing bioactive metabolites clearly associated with overall hive health, including protection against harmful microbes and contributes to the detoxification processes [34,35]. Different bacterial genera are observed in bee bread, with variation in the relative abundance of dominant taxa in the different layers of the product and types of ecosystems [30]. Regarding the main bacterial genera found in bee bread, lactobacilli appear to be the most abundant, but representatives of the genera *Bifidobacterium*, *Pseudomonas*, *Serratia*, *Bacillus*, *Sphingomonas*, *Fructobacillus*, and *Enterococcus* have been also found, with *Apb. kunkeei* being among the most common species found in bee food [30,36] (Table 1).

Depending on factors such as pollen and nectar sources, contact between bees, surrounding environment, pollination landscapes, and other variables already mentioned, there are significant variations between the main bacterial phylotypes dominant in bee microbiota, with many microbial species being endemic to the digestive tract of adult honeybees and other bee species, independent of the seasons and nutritional factors, with genera such as *Lactobacillus* (term used in the context of the significance from before changes to taxonomy in 2020 [25]) and *Bifidobacterium* appearing among the main microbial taxa, including in queen bees [8,18,32,37]. Indeed, bees and LAB have evolved in correlation with each other, and different genera of LAB are housed in the GIT compartments of bees, in their by-products, and in their hives [33,38]. LAB inhabits a niche where nutrients are available for their needs, and, on the other hand, the intestinal tract of bees is protected from harmful microorganisms by a variety of antimicrobials produced by LAB [38].

## 3. Other Bee Species Microbiota

Social bees, including honeybees, bumblebees, and stingless bees, seem to harbor a conserved gut microbiota. Thus, one might expect the five core phylotypes observed in the gut microbiota of *A. melifera* (*S. alvi*, *G. apicola*, *L. mellifer*/*mellis*, *L. melliventris*, and *Bifidobacterium*) to be predominant and abundant in all social bees. However, some of the designated core members are not always detected in sampled social bees [39]. Indeed, there are indications of a dynamic evolutionary context of the central members of the gut microbiota of social bees, with host-associated bacterial diversity in addition to significant phylogenetic clustering according to geographic location [40,41]. A recent genomic study suggests that the gut microbiota of social bees, although possibly derived from a common ancestor, is not strictly resultant of co-diversification with their hosts, and it is possible that the strength of host specificity varies among social bees or bacterial lineages [41].

Stingless bees are the largest group of social bees, and are pointed out as vital pollinators, with different ecosystems, social behavior, and nutritional habits clearly influencing the variations observed in their microbiota. Research indicates that the gut of different species of stingless bees can be associated with an established set of dominant bacteria, including species parts of *Acetobacter*, *Snodgrassella*, lactobacilli, *Psychrobacter*, *Pseudomonas*, and *Bifidobacterium* as accessed by high-throughput sequencing [42,43]. Interestingly, the bacterial phylotype identified as *Acetobacter*-like bacteria seems to be specific to stingless bee microbiota [40,42]. Most studies on stingless bees highlight that environmental factors are a strong modulating feature for the microbiota, and the microbial communities of these bees can vary significantly between different species. Moreover, even within communities of the same species, factors such as food preference, age, and genetics clearly influence which microbial communities become established [6,42].

Species of bumblebees are associated with a relevant conserved set of gut bacterial beneficial symbionts, ubiquitous across different species. These specific microbial inhabitants of bumblebees are related to those found in honeybees [44]. On the other side, microbiota of bumblebees and their distinct varieties and characteristics are not as extensively studied as compared to honeybees [45]. Specificity of the microbial inhabitants of the bumblebee clearly influences host immunity, nutrition, and protection against parasites. It has been suggested that, in the gut bacteria of the bumblebee, *Snodgrassella* plays a protective role in defense from trypanosomatida parasites [45]. Moreover, an investigation of bumblebees living at high elevations showed that their gut microbiota is closely associated with adaptations to their environment, influencing host energy metabolism and immune responses [46]. Study of the physiology and behavior of bumblebees needs to be related to deep and systematic evaluation of their microbiota, especially as many species of bumblebees (and all other bees) are in constant decline in recent decades as a consequence of use of different chemical agents in agricultural practices, reducing natural habitats, and general pollution (of all kinds). Studying these interactions can serve as scientific fundamentals that may help in developing working strategies to support bee populations, with clear acknowledgement of their ecological role, including their pollination role [45,46].

Regarding the microbiota of solitary bees, this is an entirely diverse topic and is a factor modulating host health and development. De Landa et al. [47] reported that the gut microbiome of solitary bees (*Xylocopa augusti*, *Eucera fervens*, and *Lasioglossum* spp.) is associated with the presence of species such as *Pseudomonas* as the major core taxa in all species analyzed, whereas other, such as lactobacilli, *Spiroplasma*, and *Sodalis*, were the second most abundant taxa. Observed microbial diversity is significantly affected by the presence of pathogens and environmental factors. Moreover, the principal pathogens recorded (based on biomolecular analysis) were *Nosema ceranae*, *Nosema bombi*, and *Crithidia bombi*. They were differently abundant in the different sampling sites/bee species. It is important to mention that De Landa et al. [47] pointed out that the most observed microbial taxa did not present clear correlation with land use, and only *Snodgrassella* and *Nocardioides* display higher abundances on less anthropized sites. Based on the performed study, De Landa et al. [47] suggested that pathogen species and their specific microbial load have strong influence on the gut microbial composition of solitary bees, where abundance of representatives from *Bifidobacterium*, *Apibacter*, *Serratia*, *Snodgrassella*, and *Sodalis* can positively or negatively correlate with the load of detected pathogens [47].

For solitary bees (*Osmia excavate*), the specificity of bacterial community architecture and diversity associated with the gut of larva, characteristics of the nest soil, and brood provision from the nest tube, are factors investigated and pointed to as crucial for the observed significant differences in microbial diversity between brood provision groups and nest soil groups [35]. Reported observations emphasize the complexity of the microbiota of solitary bees and the relevance of the potential impact that the microbial community of solitary bees can play on their health and physiology [35,47].

## 4. Lactic Acid Bacteria and Bifidobacteria Related to Bees and Their Products

Honey is a natural fermented food product. Although different bee species process and preserve honey in different ways, it is well established that LAB plays an important role in honey production [6]. The honey is produced from nectar collected by worker bees, temporarily stored in their stomachs while foraging near the hives. The honey stomach (crop) is a development of the esophagus, which can expand when collecting a large amount of nectar. When the crop of bees is full of nectar, the conditions of the environment are microaerobic, which favors the development of LAB. Placed in the hives, the nectar together with the LAB are at optimal temperature conditions—around 35 °C [48]. In the hive, the nectar is transferred from the mouths of worker bees who apply it to the wax honeycomb cells (*Apis* species) so that the excess liquid is evaporated (takes a few days) and the cells are then covered by a layer of wax [48]. In the case of stingless bees, honey preservation mainly depends on fermentation carried out by symbiotic microbes, including species of LAB, in closed honey pots [6].

In addition to the core microbiota, different taxonomically identified species of lactobacilli and bifidobacteria are found in the intestines of the various bee species, and in their by-products and hives [19,22,42,49]. LAB plays a crucial role in the conversion of nectar and pollen into honey and bee bread due to their fermentative activities [49]. *Apis* fresh honey, unlike stored dehydrated honey, presents a great diversity and concentration of viable LAB species [50]. LAB also takes part in protecting bees against pathogens, contributing to detoxification processes and to the bioactive properties of honeys, thus having probiotic activities [38,51].

Two important phyla of bacteria with probiotic properties are distinguished in the intestinal tract of bees and can also be present in their derivatives: Firmicutes and Actinobacteria. Of great importance are the genera of LAB from Firmicutes—enterococci, lactobacilli, lactococci, leuconostoc, pediococci, streptococci, and weissella, which have low content of G–C bonds [1,19,25]. Representatives of the type Actinobacteria include only species of the genus *Bifidobacterium* that contain high content of G-C bonds [52,53]. It is important to underline that not all representatives of LAB and bifidobacteria are beneficial and safe microbial species. Some bifidobacteria are associated with negative effects and causing dental complications in adults humans; moreover, some LAB, especially from the streptococci and enterococci genera, are well described as important effective and opportunistic pathogens not only for humans, but for other animals and insects, including bees [54]. Thus, bioinformatic studies which rely only on genus identification need to be commented on and discussed with appropriate skepticism, since non-appropriate conclusions can be drawn, and species-level identification needs to be performed.

LAB are important inhabitants of the intestinal tract of vertebrates, including humans, and invertebrates. Species of lactobacilli and enterococci are involved in numerous fermentation processes, being associated with several beneficial properties for the hosts and revealing interesting properties not only for the food industry but also for health. The antimicrobial potential of these bacteria includes production of compounds such as lactic acid, short-chain volatile fatty acids, and bacteriocin-like metabolites [55,56]. Antagonistic studies are usually aimed at preventing food spoilage by suppressing undesired microorganisms or pathogens (effective and opportunistic).

A large part of the microbiota from the GIT of bees and other nectar-feeding insects is represented by fructophilic LAB (FLAB), a special group of LAB of facultative anaerobic bacteria that prefers fructose in comparison to glucose as a carbon source, which is related to their need for an electron acceptor (oxygen, pyruvate, or fructose) during glucose dissimilation [57]. Thus, obligatory FLAB growth in the presence of glucose is limited or delayed compared to that on fructose, while facultative FLAB requires electron acceptors and aerobic conditions to grow. FLAB are found in niches rich in fructose, such as flowers and fruits, in addition to fermented fruit foods, including wine and cocoa beans [58]. The FLAB group remains little explored, but its specific physiological and biochemical characteristics, such as tolerance to phenolic acids and hexose metabolism, may have interesting implications for fermented foods and probiotics [59,60]. As a final metabolite, production of organic acids (predominant lactic acid and acetic acid) leads to a decrease in pH, and the acidic environment in fructobacilli habitats is important to the processes of prevention of the growth of spoilage microorganisms and enhancing the preservation and flavor of the honey-producing bees’ related products. In addition to low pH, fructobacilli contribute to the production of antimicrobial substances, such as hydrogen peroxide and bacteriocins (or other bacteriocin-like metabolites), as well as playing an important role in inhibiting the growth of pathogens, such as those that cause foulbrood diseases in honeybees and other honey-producing insects [61]. All fructophilic isolates reported by Endo et al. [62] were described as heterofermentative, but the obligate ones produce mainly lactic and acetic acid, and the facultative ones produce lactic acid, acetic acid, and ethanol, but in ratios different from those of heterofermentative LAB [62]. It is also worth mentioning that FLAB have a smaller genome than LAB, as they have a reduced number of genes involved in carbohydrate metabolism, and they appear to have suffered some degree of genetic depreciation during their adaptation to fructose-rich environments, since this group presents a total or partial deletion of the alcohol/acetaldehyde dehydrogenase gene (*adh*E) [58]. According to well-accepted knowledge for physiology of LAB, the preferable carbohydrate source is glucose, and normally is metabolized via the Embden–Meyerhof–Parnas Pathway [63] in homofermentative species, or via alternative pathways when some pentoses will be metabolized, normally observed in heterofermentative species. From an energetic point of view, glucose is one of the preferable carbon sources for win an energy by LAB [58].

The principal representatives of FLAB associated with bee environments are that from the genus *Fructobacillus* [59]. These bacteria were initially classified as species of the genus *Leuconostoc*, such as *Le. durionis*, *Le. ficulneum*, *Le. fructosum*, and *Le. pseudoficulneum* [64]. Later, based on phylogenetic position and biochemical and morphological characteristics, they were reclassified as species in the novel genus *Fructobacillus* [64,65]. *Fructobacillus* metabolic characteristics include the production of lactic acid, CO_2_, acetic acid, and traces of ethanol by hetero-enzymatic catabolism of a limited number of fructose-containing carbohydrates, with some species being osmotolerant. Part of the fructose, especially in the presence of glucose, is converted to mannitol [60]. Glucose metabolism is slow and very often ends with reduced CO_2_ production. Their growth on media such as All-Purpose Tween or MRS is considered not satisfactory, simply related to the fact that these media do not contain fructose as the main carbohydrate source [1].

The genus *Fructobacillus* currently comprises eleven species: *Fr. fructosus* (type species), *Fr. durionis*, *Fr. ficulneus*, *Fr. pseudoficulneus*, *Fr. tropaeoli*, *Fr. papyriferae*, *Fr. papyrifericola*, *Fr. broussonetiae*, *Fr. parabroussonetiae*, *Fr. cardui*, and *Fr. apis* [57]. Representatives from genus *Fructobacillus* were shown to utilize plant complex molecules such as lignin, which is a component of pollen [66]. *Fructobacillus* can also produce antimicrobial substances, such as organic acids and hydrogen peroxide, which inhibit the growth of pathogens in the hive, yet another beneficial property of these microorganisms for the health and well-being of several types of bees [67].

Genus *Fructilactobacillus*, part of the Lactobacilaceae family, represents some other FLAB [25,49]. It is not entirely clear whether these bacteria are also part of the GIT of bees, or if they proceed from the environment or beehives, as studies have shown that they were present in samples of honey but not in the bees’ GIT [49,68]. Another FLAB, part of the genus *Apilactobacillus*, *Apb. kunkeei*, was found to be among the dominant bacterial species in bees captured in different regions [14,50,54]. *Apb. kunkeei* is the only original fructophilic species currently classified in the genus *Lactobacillus* (in the meaning of the genus name before reclassification in 2020 [25]). The species was later considered to be FLAB.

Fructobacilli were first described in the late 1990s, when an Australian research group isolated and reported on a new species of LAB from wine with specific preferences for metabolizing fructose. Initially, this new species was named *Lactobacillus kunkeei*, honoring the Australian wine microbiologist Peter Kunkee, and his contribution to the study of wine-associated bacteria [58]. In this pioneering fructobacilli work, it was reported that the new species may produce organic acids and antimicrobial compounds from the fermentation of sugars (predominantly fructose) in wine [69]. In the agreement of the generated research data in 2008, taking into consideration specificity of their phylogenetic position, morphology, and biochemical characteristics, *Apb. kunkeei* and three other FLAB species that were formerly classified as *Leuconostoc* spp. were reclassified, and a novel genus *Fructobacillus* was born [70].

Before the genus *Fructobacillus* was introduced [57], representatives with fructose preferences were spread in different genus groups, part of the LAB and especially of the Leuconostocaceae family, which belongs to the order Lactobacillales. However, Endo and Okada [64] proposed definition of the genus *Fructobacillus* and later some representatives from four other genera (*Weissella*, *Oenococcus*, *Leuconostoc*, and *Lactococcus*) were accommodated to a new family, named Fructobacillaceae, which belongs to the order Fructobacillales. The distinguished characteristic of the new family Fructobacillaceae from the other families in the order Lactobacillales is the presence of a unique peptidoglycan type (A4α, L-Lys-D-Asp), the absence of the *rpo*A gene, and the presence of the *rpo*D gene [57].

*Apb. kunkeei* is commonly reported in association with honey-producing bees, honey as a product, and other fermented products, such as bee bread and nectar. It is also one of the main bacteria that live in the gut of honeybees, associated with processes of digestion, immunity, and protection from pathogens [65,71].

In 2020, Zheng et al. [25] suggested massive reclassifications of the former genus *Lactobacillus* and creation of several new genera, including reclassification and generation of a new genus *Apilactobacillus*, where the former *Lactobacillus kunkeei* was classified and received a new name *Apb. kunkeei*. Originally, the genus *Aplicactobacillus* accommodated *Apb. kunkeei*, *Aplicactobacillus apinorum*, and *Aplicactobacillus micheneri*, but it was later suggested that *Aplicactobacillus quenuiae* and *Aplicactobacillus timberlakei* can be considered part of the same genus as well, since it was shown that these species have metabolic preferences for D-fructose over D-glucose as growth substance and are able to metabolize D-glucose only if the appropriate electron acceptors are present [72].

As for bifidobacteria, *B. asteroides* is considered most probably the ancestor of the genus, which evolved together with the formation of gut core microbiota of pollinating insects. Reports emphasize consistently the isolation of *B. asteroides* from the GIT of bees and bee-derived environments, although in lower abundancies [8,73]. Moreover, recent studies have revealed a high degree of intraspecies genetic variety within this taxon and even suggested the need for some taxonomic adjustments [74,75]. Lugli et al. [73], referring to the applied metagenomic approaches in the analysis of dozens of *B. asteroides*-related genomes deposited in public repositories, pointed to intraspecies varieties. In the mentioned study, all specimens came from *A. mellifera* or *Apis cerrana*, and comparative analysis revealed the existence of eight clusters among so-called *B. asteroides*, five of which were characterized as a representative type of strain of the genus and three as supposed even new species of bifidobacteria [73]. These results were supported by observations from Chen et al. [75] and Liu et al. [35,76] and indicate that, for many years, there was most probably a systematic error in the classification of bifidobacteria strains related to bees within the taxon *B. asteroides*. High-throughput analysis made it possible to observe that bifidobacteria carry numerous genes involved in carbohydrate metabolism, including for a variety of polysaccharides [74,75]. A peculiarity of *B. asteroides* (or maybe it would be more appropriate to say *B. asteroides*-related) is its greater ability to tolerate oxygen compared to other species of bifidobacteria [77]. Furthermore, genome analyses of *B. asteroides* strains obtained from honeybee gut environments revealed its capacity for converting malic acid to lactic acid, which can be an addition to the standard metabolic fructose-6-phosphate pathway, associated with the presence of the fructose-6-phosphate phosphoketolase enzyme [77]. Such specificity can open some additional biotechnological applications for those strains, with possible applications in the wine industry, where malolactic fermentation is an essential step in the technological craft for cold regions wines [78].

## 5. Beneficial Properties of Fructobacilli—For Bees and Beyond

In 2011, Tajabadi et al. [79] reported for the first time the isolation and identification of strains and species of the former genus *Lactobacillus* from the stomach of the Asian giant honeybee—*Apis dorsata*. Lactobacilli were isolated from the bee stomach on selective medium—MRS. Isolates were Gram-stained and tested as negative for catalase reaction. Along with *Apb. kunkeei*, among the isolates was *Lactobacillus vermiforme*, which was first reported to be present in the bee stomach. From the obtained results, it was suggested that *Apb. kunkeei* (YH-15) could be considered the predominant species in the bee stomach, followed by *Lactobacillus* sp. Bma5 [79].

A year later, it was found that, in vitro and in vivo conditions, the LAB microbiota in *A. melifera* can be involved in the inhibition of an important bee pathogen—*P. larvae*—by forming a biofilm and networks of structures, like extracellular polymeric substances. Furthermore, Vasquez et al. [54] reported that there were other high-potential mechanisms involved in biofilm formation and adhesion such as synthesis of proteins, carbohydrates, enzymes, nucleic acids, lipids, and membrane-bound receptors. This inhibitory activity against the parasite indicates the potential importance of FLAB and their potential role as putative probiotics for bees. The authors [54] reported on a similar ICD microbiota in the nine *Apis* and three *Meliponini* species, which was expressed in species of the lactobacilli and bifidobacteria genera identified by rRNA sequencing, showing up to 97% similarity. From them, *Apb. kunkeei* was identified as the predominant bacterial species, representing 44% of the 750 identified bacterial isolates. The sensitivity of the ICD microbiota to two commonly used antibiotics in beekeeping—tylosin and oxytetracycline—was also established [54].

In 2015, Endo et al. [70] reported on results obtained from analysis of FLAB isolated from bees, larvae, honey, and bee pollen. The authors pointed out the isolation of a total of 66 strains using FGYP selective medium containing 1% fructose and glucose as substrates, supplemented with 0.005% cycloheximide and 0.005% NaN_3_. Culturing conditions for the experimental circumstances included incubation at 30 °C under aerobic conditions, on a shaker (200 rpm). Obtained cultures were also evaluated for growth on FGYP agar medium containing 0.5% CaCO_3_ and 1.2% agar under aerobic conditions for 2–3 days. Surprisingly, all isolates showed fructophilic characteristics and were distributed into six groups, four of which the authors identified as *Apb. kunkeei* and two as *Fr. fructosus*. One of the *Apb. kunkeei* isolates showed antibacterial activity against *M. plutonius*, and this protection was associated with the production of an antibacterial peptide or protein. The study suggests that bee products and larvae have a similar microbiota dominated by *Apb. kunkeei*, while adult bees have a more complex microbiota. The results demonstrate that bees and their products are a rich source of FLAB microbiota, which can be further investigated as a potential candidate for bee probiotic development [70].

## 6. Fructobacilli as Beneficial Microbes

There are several studies that focus on the GIT microflora of bees and on FLAB, which are discussed as potential probiotics for bee applications [79]. The fact that these specific microorganisms can produce bioactive compounds, antimicrobial peptides, and even some antibiotics needs to be regarded as a promising basis for the further systematic investigation for a large inhibitory spectrum contributor in combat against effective and opportunistic pathogens not only in bees, but for humans and other animals as well. It is also believed that these FLAB and their ability to produce bioactive products are the reason why honey has been known to mankind as an antimicrobial agent since ancient times [80]. LAB have been successfully applied as probiotics that contribute to the health of humans and various domestic and farm animals [81,82]. Since LAB are important components in the GIT, with a reported impact on the intestinal barrier mechanism [83], it is not surprising that LAB, especially FLAB, may be involved in maintaining good health of bees. Symbiosis is common in nature, where symbionts, such as commensals or mutualists, have evolved to benefit each other. Independent studies of the human microbiota have recently identified a complex symbiotic environment with over 1000 bacterial phylotypes representing more than 7000 strains [84]. The composition of this microbiota is thought to be the result of high co-evolutionary symbiosis and commensalism influenced by nutrition, physiology, and immunological factors.

In the last few decades, extensive research in the area of probiotics has resulted in generating solid data regarding LAB/FLAB and bifidobacteria as beneficial microorganisms. Representatives from these two bacterial groups were found to be involved in formation of the microbiota (or we should say “bacteriobiota”, since we are referring only to the bacterial representatives and not to other microbial groups) in the honeybee’s crop and gut, as well as in bee bread and honey. These two groups (LAB/FLAB and bifidobacteria) can act as probiotics for bees, enhancing their immune system, protecting them from pathogens, and improving their digestion and nutrition. Moreover, they can also produce antimicrobial substances, such as organic acids, hydrogen peroxide, and bacteriocins, that can inhibit the growth of harmful microorganisms in the hive and in honey [66,67,85,86,87]. Specificity of the GIT of honey-producing bees, and high levels of carbohydrates, specifically fructose, modulated an ecological environment where evolutionarily selected LAB/FLAB had the ability to metabolize fructose. Representatives from the genus *Fructobacillus* are more frequently reported to be principal bacterial species represented in honey-producing bees [57,88]. FLAB may also produce antimicrobial substances, already extensively studied in LAB, such as hydrogen peroxide and bacteriocins [89,90]. The latter are shown to be actively involved in the inhibition of pathogens; however, in relation to FLAB, these antimicrobials inhibit pathogens related to those that cause foulbrood diseases in honeybees [57,70,91].

### 6.1. Apilactobacillus kunkeei as a Beneficial Species

It was reported that *Apb. kunkeei* can inhibit *P. larvae* and *M. plutonius*, which are involved in causing American foulbrood and European foulbrood, respectively, in honeybees. Moreover, taking into consideration that *Apb. kunkeei* was one of the dominant bacteria in the honey crop and gut of honeybees, its role as a probiotic for bees, enhancing their immune system, producing vitamins, and influencing their behavior and physiology, was even more relevant [92]. Scientific interest in the beneficial properties of *Apb. kunkeei* resulted in revealing its diversity, ecology, physiology, genetics, and applications. From an academic point of view, it is relevant to mention the study conducted by Tamarit et al. [65], where the authors compared the genomes of ten different strains of *Apb. kunkeei* isolated from different bee species. The authors reported that investigated strains had a unique genome organization, where core genes are located near the terminus of replication and accessory genes located near the origin of replication. Moreover, Tamarit et al. [65] pointed out that *Apb. kunkeei* had high gene flux, most probably associated with the influence of reductive evolution and horizontal gene transfer.

In another study, Lashani et al. [93] reported antimicrobial activity of *Apb. kunkeei* against foodborne pathogens including *Listeria monocytogenes*, *Shigella flexneri*, *Staphylococcus aureus*, *Salmonella enteritidis*, Enteropathogenic *Escherichia coli*, *E. coli* O157 H7, and *Bacillus cereus*. Zendo et al. [89] reported that *Apb. kunkeei* produced novel bacteriocins, named kunkeecin. Ebrahimi characterized in vitro *Apb. kunkeei* ENH01 regarding to its antibacterial and antifungal properties on *E. coli* and *Asp. niger*, respectively [71]. Moreover, according to performed in situ inhibitory experiments, it was shown that *Apb. kunkeei* ENH01 cell-free supernatant presented an inhibitory effect on *Candida albicans* up to almost 77% inhibition. Furthermore, the presence of three antibacterial peptides was associated with a studied *Apb. kunkeei* ENH01 strain, according to the performed liquid chromatography mass spectrometry (LC/MS) assay. Anti-mycotoxigenic properties of *Apb. kunkeei* ENH01, recorded on viable and heat-killed cells, were also observed and validated by analyzing against total aflatoxins according to HPLC-based analysis [71].

Teame et al. [94] reported on probiotics and postbiotic properties of *Apb. kunkeei* and the role of produced organic acids, such as lactic acid and acetic acid, that lower pH and increase the acidity of honey and nectar, associated with prevention of the growth of spoilage microorganisms and enhancing the preservation and flavor of the products. The authors also reported on production of antimicrobial compounds (hydrogen peroxide, ethanol, and bacteriocins) involve in the growth inhibition of *P. larvae* and *M. plutonius*, pathogens associated with American foulbrood and European foulbrood in honeybees.

Chen et al. [75] informed on antimicrobial properties of *Apb. kunkeei* against carbapenem-resistant Enterobacteriaceae (CRE), a multidrug-resistant bacteria associated with serious infections in humans and animals. In the mentioned study [75], different lactobacilli, including *Apb. kunkeei*, were evaluated versus 12 CRE strains. Moreover, it was pointed out that cell-free supernatant from *Apb. kunkeei* showed the highest antimicrobial activity against tested CRE, with a minimum inhibitory concentration (MIC) of 0.5% and a bactericidal effect at 2%. In addition, the authors also analyzed *Apb. kunkeei* cell-free supernatant obtained by LC/MS and reported that, in addition to expected lactic acid, acetic acid, and ethanol, two bacteriocins, namely, kunkeecin A and kunkeecin B, were part of the antimicrobial cocktail.

The type strain *Apb. kunkeei* YH-15, originally isolated and reported by Huang et al. [95], was investigated for its beneficial properties as well. The authors reported on *Apb. kunkeei* YH-15, isolated from damaged grapes in wine production from California, USA, and it was observed that that strain was able to inhibit yeasts and, regarding these properties, it was considered a spoilage organism of wine production [96]. The authors also suggested that, most probably, the origin of that strain of *Apb. kunkeei* needs to be associated with honeybees themselves, since while searching for some sugar sources, they transferred the strain to the grape [95,97]. *Apb. kunkeei* can ferment the sugars present in nectar and, because of metabolic activity, can produce organic acids, such as lactic acid and acetic acid, typical products for LAB. As a consequence, it can prevent the growth of spoilage microorganisms and enhance the preservation and flavor of the honey. Moreover, it was reported that *Apb. kunkeei*, as well as several other LAB/FLAB, can produce numerous antimicrobial compounds, including hydrogen peroxide, ethanol, and bacteriocins, that can inhibit the growth of pathogens. It was suggested that antimicrobials produced by *Apb. kunkeei* have an important role in inhibition of honeybee-associated pathogens, including *P. larvae* and *M. plutonius*, which cause American foulbrood and European foulbrood, respectively, in honeybees [66,67].

A specificity of the metabolism of *Apb. kunkeei* is the fact that this FLAB has metabolic preferences for fructose as its main carbon and energy source. However, it can also use glucose, sucrose, and mannitol, but not other sugars, such as lactose or maltose. It can produce lactic acid, acetic acid, ethanol, and other organic acids from the fermentation of sugars, which can lower the pH and increase the acidity of the environment. This can prevent the growth of spoilage microorganisms and enhance the preservation and flavor of honey and other products [98]. As with several other LAB, *Apb. kunkeei* is an aerotolerant organism. It was reported that some strains belonging to the species can produce bacteriocins (antimicrobial peptides) with activity against *P. larvae* and *M. plutonius* [98]. Some other beneficial properties were also associated with the presence of *Apb. kunkeei* in bees’ guts and their hives, including enhancing their immune system and modulating their immune responses, production of vitamins (biotin and riboflavin), and playing an essential role in bioavailability of iron, all of them essential for the growth and development of bees. *Apb. kunkeei* is associated with normal functions of bees and their behavior and physiology, such as foraging, learning, memory, and stress resistance, playing an essential role in processing the complex carbohydrates and proteins in their diet, such as pollen, nectar, and bee bread [98].

Moreover, it was suggested that *Apb. kunkeei* can be considered a potential human probiotic, as some strains from the species have positive effects on the intestinal microbiota and the immune system, involved in the inhibition processes of pathogenic bacteria (*E. coli*, *Salmonella typhimurium*, and *Helicobacter pylori*) in the human gut, whereas it can also be involved in the modulation of the production of cytokines and regulation of the processes of inflammation and immunity. In addition to *Apb. kunkeei*, other microbial species associated with healthy bees were evaluated regarding their probiotic properties for bees, humans, and other animals [99].

### 6.2. Other Bees Associate Species as Beneficial Organisms

Species such as *G. apicola*, *Sn. alvi*, *Bartonella apis*, and *Frischella perrara* are some examples reported to be part of the core gut microbiota of honeybees. It was reported that they are essential for the digestion and nutrition of bees and are essential in the assimilation of complex carbohydrates and proteins in bees’ diet. The mentioned species are involved in the modulation of the immune system of bees, in processes of protection from pathogens and parasites, and can influence bees’ behavior and physiology. For example, it was shown that *G. apicola* contributes by production of vitamins (biotin and riboflavin), essential for the growth and development of bees. Strains of *Sn. alvi* were reported to be producers of antimicrobial peptides (alveicin), which actively contribute to the killing of *P. larvae*. Moreover, *Ba. apis* can produce siderophores, such as enterobactin, that can bind and transport iron, which is an essential nutrient for bees. *Fri. perrara* can induce the production of antimicrobial peptides, such as defensin-1, in bees [66,100].

The complex gut microbiota of bees is associated with numerous physiological functions, including digestion, nutrition, immunity, and behavior. However, different types of bees, and even differences in environmental habitats, can influence the diversity of the gut microbiota. However, some core groups and species are predominant. It was shown that the gut microbiota of bees is mainly composed of bacterial species, with some yeasts and fungi also present [45]. Considering that bees are social insects with well-established hierarchy, the gut microbiota of bees is transmitted socially, meaning that bees acquire their microbes from their nestmates, food sources, and environment [74]. However, the gut microbiota of bees is host-specific, well adapted to the particular species and subspecies of bees that they inhabit, but at the same time relatively stable, with not much change over time or across different locations [74,100]. The contribution of the gut microbiota of bees is beneficial and can provide various advantages to bees, such as enhancing their immune system, protecting them from pathogens and parasites, producing vitamins and other nutrients, and influencing their behavior and physiology [42,74]. Some of the most well-studied gut microbiota of bees are those of honeybees and bumblebees, which belong to the same family (Apidae) and it was even found that they can share some common gut bacteria, even inhabiting different geographical regions. At the same time, some specificities, and differences between them can be mapped. As previously mentioned, the gut microbiota of honeybees in most of the reports is associated with consistency of eight core bacterial taxa: *Sn. alvi*, *G. apicola*, bifidobacteria, *L. melliventris* clade, *L. mellifer* clade, *Fri. perrara*, and *Ba. apis* [74,101]. The gut microbiota of bumblebees consists of only five core bacterial taxa: *Sn. alvi*, *G. apicola*, bifidobacteria, *L. melliventris* clade, *L. mellifer* clade [42]. Moreover, the gut microbiota of honeybees and bumblebees differ not only on principal groups of bacterial genera, but in their strain-level diversity, with the presence of different variants of the same bacterial species. It was reported that *Sn. alvi* has more strain variety in honeybees than in bumblebees, while representatives from bifidobacteria have more strain variety in bumblebees than in honeybees [42]. The role of that species in honeybees and bumblebees is also different regarding their functional traits, as they can have different abilities and roles in the bee gut. For instance, *Sn. alvi* is associated with production of antimicrobial peptides and siderophores in honeybees, but not in bumblebees.

### 6.3. Bifidobacteria as a Beneficial Species

Bifidobacteria are associated with degradation of lignin and polysaccharides in bumblebees, but do not have this role in honeybees [42]. Moreover, the gut microbiota of all kinds of bees are essential and clearly related to the health and survival of these pollinators. Moreover, improvement of immune functions, protecting them from pathogens and parasites, and modulating their immune responses, producing vitamins and other nutrients, such as biotin, riboflavin, and iron, and influencing their behavior and physiology, such as foraging, learning, memory, and stress resistance. Helping them process the complex carbohydrates and proteins in their diet, such as pollen, nectar, and bee bread. Producing antimicrobial substances, such as organic acids, hydrogen peroxide, ethanol, bacteriocins, and antimicrobial peptides, that can inhibit the growth of harmful microorganisms in the hive and in honey [102]. These are just some functions associated with variety in the gut microbiota.

Even if it is well stated that the gut microbiota of bees is relatively stable [102], environmental conditions can play a negative role in the numbers and variety of the microbial species representing the gut microbiota of bees. Some of the environmental stress factors are nutritional fluctuations, artificial feeding supplements, agrochemicals, hive treatments, radio and magnetic fields, and pathogens, and can play significant role in bees’ health and even survival. Consequently, these stressors can cause strong shifts in the composition, diversity, and function of the gut microbiota, which may in turn affect the health and performance of bees [102]. Therefore, it is important to monitor and maintain the health and diversity of the gut microbiota of bees, as they are vital for the well-being of these pollinators.

### 6.4. Some Other Fructobacilli as Beneficial Species

Some of the species belonging to the genus *Fructobacillus*, isolated from bee bread, brood cells, and larval gut, were suggested as probiotics regarding their ability to utilize the plant complex molecule lignin, which is a component of pollen, thus beginning the breakdown of this important high-protein plant-derived food for bees. Some representatives from the genus *Fructobacillus* were also associated with production of antimicrobial substances, such as organic acids and hydrogen peroxide, that can inhibit the growth of pathogens in the hive [103]. *Bar. apis* belongs to the core gut microbiota of honeybees. It was reported that *Bar. apis* strains can produce siderophores, such as enterobactin, that can bind and transport iron, which is an essential nutrient for bees. Strains from this species can also be involved in the modulation of the immune system of bees and protect them from pathogens and parasites [66]. Microorganisms with probiotic properties can help honeybees with various functions, such as digestion, nutrition, immunity, and protection from pathogens. These benefits include enhancing their immune system, protecting them from pathogens and parasites, and modulating their immune responses. It was shown that *Apb. kunkeei* can trigger the upregulation of immune gene expressions and reduce prevalence, indicating that immune priming underlies the microbial protective effect and can influence their behavior and physiology, such as foraging, learning, memory, and stress resistance [103]. For example, *Fructobacillus*, a genus of bacteria that is isolated from bee bread, brood cells, and larval gut, can influence the foraging behavior of honeybees by affecting their sucrose responsiveness [103].

In addition, beneficial microbes in bees can improve their growth and development, since specific probiotics can help them digest and absorb nutrients, such as protein and calcium, from their diet of pollen, nectar, and bee bread. Some probiotic strains can enhance their foraging behavior and performance, as probiotics can affect their sucrose responsiveness, learning, memory, and stress resistance, and are associated with increasing their honey production and quality, as probiotics can ferment the sugars in nectar and produce organic acids, such as lactic acid and acetic acid, that lower the pH and increase the acidity of honey. This can prevent the growth of spoilage microorganisms and enhance the preservation and flavor of the honey. Application of probiotics can be responsible for a reduction in colony losses and mortality, as probiotics can protect them from various diseases, including American foulbrood, European foulbrood, chalkbrood, nosema, and varroa mites, by producing antimicrobial substances, such as hydrogen peroxide, ethanol, bacteriocins, and other antimicrobial peptides, that can inhibit the growth of pathogens and parasites [85].

## 7. Final Remarks

Yes, we clearly acknowledge the ecological and economical role of different varieties of bees, and their decline in the last decade. It is a well-stated fact that disbalance of the ecological equilibrium associated with bees’ decline can have significant negative consequences. In this context, development research and application in probiotics for bees, particularly honeybees, can be one of the actions that needs to be considered. Some examples in this direction are a probiotic supplement BioPatty, developed with the aim to enhance honeybee health. Application of this product aims to boost bees’ immune response and improve their defense mechanisms and combat versus pathogens, such as bacterial pathogens causing American foulbrood. Based on the performed assays, it is suggested that the use of these probiotics can result in a 99% reduction in pathogen load and a significant increase in bee survival rates [104]. Moreover, while most of the investigations and development of probiotics are for honeybees, studies need to be extended (and applied) to other bee species, including stingless bees, solitary bees, bumblebees, and other varieties, spread all over the world in different ecosystems. The main concept is not to generate “super-species” of bees, but to naturally strengthen bees’ gut microbiota, and via this improve their health and ability to combat against diseases with a goal to prevent colony collapse.

Implementing probiotics in bees on a large scale is a delicate process and presents specific challenges, including appropriate beneficial strain selection, comprising the process of identifying and developing relevant probiotic strains that will be effective across various bee species and environmental conditions. Delivery methods are an additional technological challenge and will need to guarantee that the applied probiotics will be supplied in a form that bees will be able to consume and simply will survive in the hive environment. Applying the microorganisms into the environment need to be according to the sanitary regulations. Regulatory hurdles can be relevant points to be considered in the applications of probiotics for bees. Specific legal and regulatory frameworks that govern the use of probiotics in agriculture and beekeeping need to be respected. We all know the role of bees; however, cost is always a factor to be considered. Developing affordable cost-effective production and distribution methods are important steps for further probiotic affordable applications for beekeepers. Furthermore, all these are clearly related to conducting extensive research to understand the long-term effects of probiotics on bee health, genetics, and behavior. All these require coordinated efforts from different researchers, industry, and regulatory bodies to guarantee that probiotics can be safely and successfully applied to support bee populations [22].

Probiotics developed by research institutions will help to improve the benefits for bee health. Probiotics can be applied as supplemental feeding, by adding probiotics to bees’ food, such as sugar syrup or pollen patties, specifically through times of stress or when natural forage is limited. However, this needs to be clearly according to regular administration recommendations, providing probiotics on a regular schedule to maintain a healthy gut microbiota in bees. In addition, post-application of probiotics (even validated by the appropriate research tests), monitoring bee colony health needs to be performed, keeping track of the health and productivity of the colonies to assess the effectiveness of the probiotics [22,105].

However, application of probiotics needs to be a responsible process, since overusing (abusing) of probiotics in bee hives can lead to several risks, including resulting in the disruption of microbial balance as a consequence of excessive probiotics, which can disrupt the hive’s homeostasis, potentially leading to unforeseen health issues. In some cases, resistance development can result from inappropriate applications of probiotics and as a consequence some pathogens may develop resistance to the beneficial bacteria, making them less effective over time. A resource competition can be a result of the fact that probiotics might compete with bees for resources within the hive, such as food or space. Unintended effects of probiotics (changes in the host microbial balance) on bee behavior related to specific changes in the gut microbiota could potentially alter bee behavior, affecting foraging patterns or communication. There are also regulatory and environmental concerns regarding the release of non-native organisms into the environment, as well as potential impacts on local ecosystems [104,106].

With the aim to reduce the risks of applications of probiotics and to provide appropriate, effective solution that can work effectively in bee protection, current research on addressing the risks associated with bee probiotics is focused on several key areas including evaluation of their efficacy and beneficial outcomes, where research projects focus on testing for the effectiveness of commercially sold probiotics on restoring the gut microbiota of honeybees after antibiotic treatment [107]. In addition, researchers are evaluating the theoretical risks of the application of probiotics, taking into consideration factors such as systemic infections, deleterious metabolic activities, and excessive immune stimulation [108]. Application of probiotics (not only in bees) can have influence on microbial diversity. There is ongoing research to characterize the microbial diversity associated with honeybees and the use of probiotic symbionts to maintain honeybee fitness [22]. Appropriate safety protocols need to be a priority. Investigations into the safety of probiotics are being conducted (and will be conducted), including potential side effects and the risk of gene transfer [109]. In fact, all this is with a clear aim that these research efforts aim is to ensure that applied probiotics can be resulted on safe and beneficial contribution associated with effective support to bee health and mitigate the risks of overuse.

## Figures and Tables

**Table 1:** Microbial diversity of the bee’s microbiota, with focus on phylum and genus representation and some species examples.

Phylum	Genus	Species
Firmicutes	*Bombilactobacillus*	*Bombilactobacillus mellifer* *Bombilactobacillus mellis*
	*Lactobacillus*	*Lactobacillus melliventris*
	*Apilactobacillus*	*Apilactobacillus kunkeei* *Apilactobacillus apinorum* *Apilactobacillus micheneri* *Apilactobacillus quenuiae* *Apilactobacillus timberlakei*
	*Fructobacillus*	*Fructobacillus fructosus* *Fructobacillus durionis* *Fructobacillus ficulneus* *Fructobacillus pseudoficulneus* *Fructobacillus tropaeoli* *Fructobacillus papyriferae* *Fructobacillus papyrifericola* *Fructobacillus broussonetiae* *Fructobacillus parabroussonetiae* *Fructobacillus cardui* *Fructobacillus apis*
	*Leuconostoc*	*Leuconostoc durionis* *Leuconostoc ficulneum* *Leuconostoc fructosum* *Leuconostoc pseudoficulneum*
	*Pediococcus*	
	*Streptococcus*	
	*Weissella*	
	*Enterococcus*	
	*Lactococcus*	
Actinobacteria	*Bifidobacterium*	*Bifidobacterium asteroides*
Pseudomonadota	*Bombella*	*Bombella apis*
	*Snodgrassella*	*Snodgrassella alvi*
	*Pseudomonas*	
	*Sphingomonas*	
Bacilota	*Bacillus*	
Proteobacteria	*Gilliamella*	*Gilliamella apicola*
Proteobacteria	*Serratia*	
Bacteroidetes		
Cyanobacteria		
Verrucomicrobia		
Acidobacteria		
Spirochaetes		
Planctomycetes		

## Data Availability

No new data were created.
